# Synergistically Enhancing Capacitive Performance of Ti_3_C_2_T_x_ MXene via Building Hierarchical Structure of TiO_2_ Nanowire/MXene Composites and Utilizing Iron-Ion-Based Redox-Active Electrolytes

**DOI:** 10.3390/nano16110671

**Published:** 2026-05-27

**Authors:** Xiaohan Wang, Xusheng Du

**Affiliations:** Institute of Advanced Wear & Corrosion Resistant and Functional Materials, School of Chemistry and Materials, Jinan University, Guangzhou 510632, China

**Keywords:** Ti_3_AlC_2_, Ti_3_C_2_T_x_, MXene, supercapacitor, redox-active electrolyte

## Abstract

In this work, a strategy for synergistic regulation of the Ti_3_C_2_T_x_ surface structure and redox activity of the electrolyte has been proposed. The surface modification of MXene was achieved via KOH treatment. Meanwhile, to cooperate with the surface-modified MXene electrode materials, Fe^3+^/Fe^2+^ was introduced into its common H_2_SO_4_ electrolyte to operate as a redox-active electrolyte for the first time. The results indicate that alkali treatment not only effectively reduces the amount of fluorine-terminal groups on the MXene surface but also forms in situ TiO_2_ nanowires on its surface, thereby forming a unique hierarchical structure for facilitating the electrochemical reaction. Further utilization of the Fe^2+^/Fe^3+^ redox-active electrolyte introduced additional pseudocapacitive reactions at the electrode/electrolyte interface, significantly enhancing the capacitive performance of the system. This synergistic effect of both the hierarchical 1D TiO_2_/MXene composite electrode materials and the redox-active electrolyte resulted in a substantial increase in specific capacitance from 78.17 F g^−1^ to 655.54 F g^−1^ at a current density of 10 Ag^−1^. The reaction kinetics of the electrochemical systems were studied, along with their energy storage mechanism. It is revealed that there is a transition of the energy storage mechanism from being dominated almost solely by diffusion control to collaborative diffusion and surface reactions in the synergistic electrode/electrolyte system, and the corresponding equivalent circuit has evolved from the single-interface model to a dual-interface model. This work has demonstrated that the proposed synergistic strategy can effectively enhance the capacitive performance of the MXene energy storage system and can be applied to other electrochemical systems.

## 1. Introduction

Driven by the escalating demand for high-performance energy storage technologies, supercapacitors have attracted intense research interest due to their rapid charge–discharge kinetics and remarkable power densities. Nevertheless, traditional carbonaceous and metal oxide electrodes are frequently hindered by insufficient specific capacitance and inferior electrical conductivity, respectively. To circumvent these trade-offs, developing alternative electrode architectures that simultaneously possess high electronic conductivity and robust electrochemical reactivity has become a paramount objective. In this regard, two-dimensional (2D) transition metal carbides/nitrides, commonly designated as MXenes, have emerged as highly promising candidates [1,2,3]. Specifically, Ti_3_C_2_T_x_ stands as the most widely investigated archetype, benefiting from its metallic-like conductivity, excellent hydrophilicity, abundant surface chemistry, tunable interlayer spacing, and ready synthetic availability [4]. Consequently, Ti_3_C_2_T_x_ holds immense potential across diverse fields, encompassing not only electrochemical energy storage [5,6,7,8] but also chemical and biological sensing [9,10], lubrication [11], and electrocatalysis [12,13].

Tailoring the physicochemical properties of electrode matrices represents a universal approach to bolstering their surface-active sites. Among various modification protocols, alkaline etching is recognized as a highly cost-effective and straightforward strategy to engineer the surface terminations and structural galleries of MXenes. Previous literature indicates that alkali exposure substantially strips the fluorine (-F) terminal groups from the Ti_3_C_2_T_x_ surface while enriching hydroxyl (-OH) functionalities, a process typically accompanied by interlayer expansion that enhances both active-site exposure and ion accessibility [14]. Furthermore, Chen et al. demonstrated that such alkali-induced modification effectively mitigates the self-restacking of MXene flakes, thereby accelerating electrolyte ion transport and upgrading capacitive delivery [15]. This alkaline regime has also been reported to multiply surface-active centers and lower charge-transfer impedance, ultimately leading to augmented rate kinetics and capacitance [16]. Collectively, these insights indicate that alkali treatment optimizes the layered topology of MXenes while dynamically modulating surface terminations to enhance interfacial charge transfer. Notably, under strongly alkaline conditions, superficial Ti atoms can undergo partial oxidation to yield TiO_2_ or Ti-O active domains, which introduce supplemental pseudocapacitive pathways and catalyze interfacial redox processes [17].

Parallel to electrode engineering, manipulating the electrolyte configuration offers another pivotal avenue to upgrade energy storage performance, given that the electrode–electrolyte interaction inherently dictates system-level behavior. Traditional aqueous electrolytes rely predominantly on electrostatic double-layer capacitance (EDLC), which intrinsically caps the energy density of the configuration. Incorporating redox-active species into the electrolyte can breach this bottleneck by introducing external pseudocapacitance via highly reversible Faradaic reactions, thereby driving up both specific capacitance and energy density [18,19]. Among candidate redox couples, the Fe^3+^/Fe^2+^ system is particularly enticing due to its outstanding electrochemical reversibility and rapid reaction kinetics. Incorporating Fe^3+^ species can trigger supplemental interfacial Faradaic processes at the electrode–electrolyte boundary, markedly enhancing the overall charge-storage capacity [20,21]. For instance, carbon nanofiber/stainless steel networks have been shown to deliver an exceptional specific capacitance of 2988 F/g when operated within a 0.8 M Fe^2+/3+^-H_2_SO_4_ electrolyte [20].

MXene-based electrodes are typically paired with sulfuric acid (H_2_SO_4_) electrolytic environments. The iron-based couple (Fe^2+^/Fe^3+^), derived from an earth-abundant and highly economical transition metal, exhibits exceptional chemical stability at high concentrations (up to 0.8 M) within acidic media [20]. The highly reversible redox chemistry of iron ions allows them to serve a dual purpose within the device [21]: they concurrently regulate the ionic conductivity and mechanical integrity of the bulk electrolyte, while boosting the overall energy storage capacity via reversible Faradaic reactions. Given that MXenes possess graphene-like 2D topologies paired with superior metallic conductivity, pairing an alkali-activated MXene matrix with an iron-based redox electrolyte is expected to unlock powerful synergistic effects, substantially maximizing the system’s capacitive metrics. Surprisingly, however, systematic investigations into such integrated electrochemical configurations remain remarkably scarce to date.

In this work, MXene will be surface-modified via alkaline treatment and the resulting products will be characterized. The effect of the alkaline treatment of MXene on its electrochemical capacitive properties will be studied. Moreover, an iron-ion redox-active electrolyte will be prepared by adding ferric sulfate and ferrous sulfate to a conventional sulfuric acid electrolyte, and the electrochemical behavior of the surface-modified MXene in the redox-active electrolyte will be investigated comprehensively with a range of analytical techniques. Additionally, the effect of the concentration of iron ions in the electrolyte on the electrochemical performance of the MXene-based electrode will be investigated. Furthermore, the reaction kinetics of modified MXene in the redox-active electrolytes and the corresponding energy storage mechanism will be revealed.

## 2. Materials and Methods

Commercial Ti_3_AlC_2_ MAX powder (400 mesh, >99.99% purity) and lithium fluoride (LiF) were secured from Foshan Xinxi Technology Co., Ltd. (Foshan, China). Potassium hydroxide (KOH) was supplied by Shanghai Macklin Biochemical Co., Ltd. (Shanghai, China). Concentrated hydrochloric acid (HCl) was obtained from Guangdong Guangshi Reagent Technology Co., Ltd. (Guangdong, China) All chemicals were utilized in their as-received state without supplementary purification.

A typical approach was employed to synthesize Ti_3_C_2_T_x_ flakes using a LiF/HCl etchant. In a 100 mL PTFE reactor, 3.2 g of LiF was dissolved in a premixed acid solution comprising 30 mL of concentrated HCl and 10 mL of deionized (DI) water under continuous stirring for 15 min. Subsequently, 2 g of Ti_3_AlC_2_ powder was introduced portion-wise into the etchant, and the reaction was maintained at 40 °C for 48 h. The acidic slurry was then isolated via centrifugation (5000 rpm, 5 min per cycle) and repeatedly rinsed with DI water until a neutral pH of ~6 was achieved. To delaminate the multilayered framework, the suspension underwent a 1 h sonication, followed by a 30 min centrifugation at 5000 rpm to harvest the colloidal Ti_3_C_2_T_x_ flakes from the supernatant.

For the surface alkali-tailoring process, 0.3 g of the collected MXene was dispersed into 20 mL of a 10 M KOH aqueous solution. The alkaline treatment was carried out at 65 °C under vigorous stirring for either 12 h or 24 h. The resulting products were sequentially centrifuged, washed with DI water to remove residual alkali, and the target functionalized fragments were gathered from the supernatant, which were designated as AT-MXene-A (12 h) and AT-MXene-B (24 h), respectively.

The redox-active electrolytes were formulated by co-dissolving equimolar quantities of ferric sulfate (Fe_2_(SO_4_)_3_) and ferrous sulfate (FeSO_4_) into a baseline matrix of 1 M H_2_SO_4_ (50 mL). Depending on the salt concentrations, the mixed electrolytes were classified into three groups: a solution with 0.8 M Fe^3+^ and 0.8 M Fe^2+^ was designated as 8Fe; similarly, the concentrations of 0.4 M and 0.2 M for both iron ions were denoted as 4Fe and 2Fe, respectively.

The crystalline phases and structural attributes of the samples were examined using an X-ray diffractometer (Rigaku Ultima IV, Tokyo, Japan) equipped with a Cu Kα radiation source (λ = 1.54 Å), scanning from 4° to 80° at a constant velocity of 5°/min. Raman fingerprinting was accomplished on a LabRAM HR Evolution spectrometer (HORIBA Scientific, Paris, France). Ultraviolet-visible (UV-Vis) absorption behavior was recorded using a Shanghai I&E L6S Plus spectrophotometer (Shanghai, China) within a baseline-calibrated DI water medium, employing a data acquisition interval of 1.0 nm and a scan speed of 100 nm/min.

All electrochemical tests were performed on a Gamry 1010E electrochemical workstation utilizing a conventional three-electrode configuration. A Pt plate served as the counter electrode, and a saturated calomel electrode (SCE) was employed as the reference electrode. To construct the working electrode, a homogeneous slurry containing the MXene active material, acetylene black, and a polytetrafluoroethylene (PTFE) binder in a weight ratio of 80:10:10 was fabricated. The testing electrolytes consisted of either a pristine 1.0 M H_2_SO_4_ solution or 1.0 M H_2_SO_4_ enriched with varying concentrations of Fe^2+^/Fe^3+^ ions. Electrochemical impedance spectroscopy (EIS) profiles were swept across a frequency spectrum from 100 kHz to 0.01 Hz under an AC perturbation amplitude of 5 mV, and the impedance profiles were fitted via ZView 3.0 software. The gravimetric specific capacitance values were calculated from the acquired galvanostatic charge–discharge (GCD) and cyclic voltammetry (CV) profiles based on the following expressions:

(1) $$C=\frac{I∆t}{m∆V}$$

(2) $$C=\frac{1}{2mv∆V}\int_{\varphi i}^{\varphi \lambda }I(V)dV$$

where ΔV is the potential difference, m is the mass of the active material, C is the specific capacity, I is the discharge current, and Δt is the discharge time.

The gravimetric energy density (E, Wh kg^−1^) is then calculated by

(3) $$E=\frac{C{∆V}^{2}}{2}$$

The gravimetric power density (P, W kg^−1^) can be calculated by

(4) P=3600E/∆t

## 3. Results and Discussion

### 3.1. Regulation of MXene Structure via KOH Treatment

**Figure 1:**
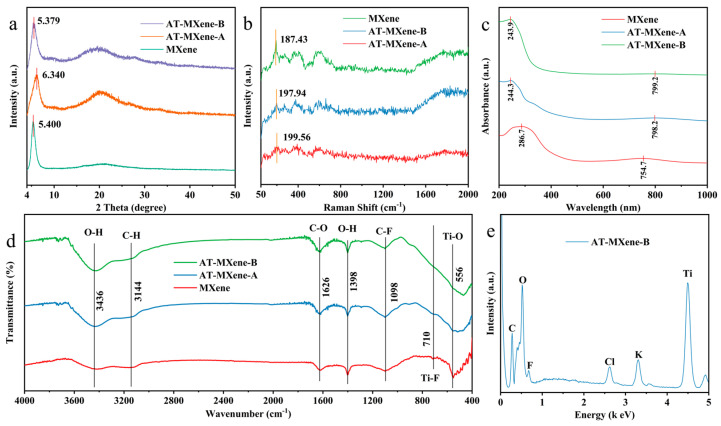
(**a**) XRD patterns, (**b**) Raman spectra, (**c**) UV-Vis spectra, and (**d**) FTIR spectra of MXene, AT-MXene-A, and AT-MXene-B; (**e**) EDS elemental spectra of AT-MXene-B.

Ti_3_C_2_T_x_ MXene was treated in a KOH solution for different durations to modulate its surface chemical structures. As shown in Figure 1a, both KOH-treated samples exhibit typical (002) diffraction peaks in the low-angle region. The (002) peak of the pristine Ti_3_C_2_T_x_ MXene is located at 5.40°. After KOH treatment for 12 h, the (002) peak of AT-MXene-A shifts slightly to 6.34°, indicating a decrease in the interlayer spacing of the sample. This could be due to the so-called ‘chaotropic cation intercalation mechanism’ [7], which relates to the intercalation of hydrophobic potassium cations, the volume repulsive effect and strong interaction between MXene nanosheets and K^+^. Specifically, for the treatment for a longer time (24 h), the (002) peak of AT-MXene-B shifted to a lower degree, suggesting an increasing interlayer spacing, which could be due to the intercalation of more potassium cations and extensive changes in surface terminal groups on MXene surface. These results indicate that KOH treatment not only regulates the interlayer spacing of MXene but also alters its chemical composition. This is similar to the findings on the structural regulation of MXene through alkaline treatment [17].

As shown in Figure 1b, all three MXene samples display characteristic Ti-C vibrational peaks in the low wavenumber region (100–300 cm^−1^) in their Raman spectra, similar to other reports [22]. However, a significant blue shift in the peak positions is observed after the alkali treatment. The peak position for pristine Ti_3_C_2_T_x_ MXene is located at 187.43 cm^−1^, while those for AT-MXene-B and AT-MXene-A shift to 197.94 cm^−1^ and 199.56 cm^−1^, respectively. Such a peak shift indicates a change in the local chemical environment of Ti-C bond, which is typically associated with surface group transformations (e.g., from -F to -O or -OH) and changes in lattice stress [23]. These changes suggest that KOH treatment modulates the surface chemical state of MXene, potentially enhancing its electrochemical activity and pseudocapacitive behaviors. In its UV absorption spectrum (Figure 1c), pristine Ti_3_C_2_T_x_ MXene displays a distinct UV absorption peak at 286.7 nm, accompanied by a broad near-infrared absorption peak near 754.7 nm, which are characteristic absorption features of Ti_3_C_2_T_x_ MXene. As suggested in previous studies [24,25], the absorption peak that occurs at approximately 240–290 nm is commonly ascribed to electronic transitions associated with the Ti-C bond. In contrast, the broad peak that is observed near 800 nm is attributed to the localized surface plasmon resonance (LSPR) absorption, which is the result of the oscillation of free electrons in MXene. Consequently, these two characteristic peaks are indicative of the structural state of MXene and the concentration of conductive electrons, respectively. After a 12 h alkali treatment, a significant shift from 286.7 nm for pristine MXene to 244.3 nm for AT-MXene-A sample was observed. Further extension of the alkali treatment time to 24 h resulted in a significant shift to 243.9 nm, as seen in the UV absorption peak of AT-MXene-B. Such a shift could be attributed to the surface oxidation of MXene. In the alkaline environment, active titanium (Ti) sites on the Ti_3_C_2_T_x_ surface tend to react with hydroxyl (OH^−^) ions and/or dissolved oxygen, resulting in the formation of Ti-O bonds or even TiO_2_. Changes in the near-infrared absorption peaks further confirm the oxidation of MXene after the alkaline treatment. In the spectrum of AT-MXene-A, a peak appears at 798.2 nm, showing a significant red shift compared to that of pristine MXene (754.7 nm). Meanwhile, that of AT-MXene-B shifts even further to 799.2 nm, accompanied by a significant decrease in peak intensity, indicating a marked decrease in free electron concentration and a weakening of metallic properties. These changes could be ascribed to the formation of TiO_x_/TiO_2_ on the MXene surface, which alters the electronic structure of the Ti-C framework and reduces the concentration of free electrons. FTIR spectroscopy is a viable analytical method for the study of F-terminal groups within Ti_3_C_2_T_x_ [26]. Subsequent to KOH treatment, a significant alteration in the Ti-F bonds was observed. As shown in Figure 1d, the Ti-F bending vibration peak for pristine Ti_3_C_2_T_x_ is located at 710 cm^−1^ and it becomes too weak to be defined in both AT-MXene-A and AT-MXene-B, confirming the removal of F-terminal groups.

**Figure 2:**
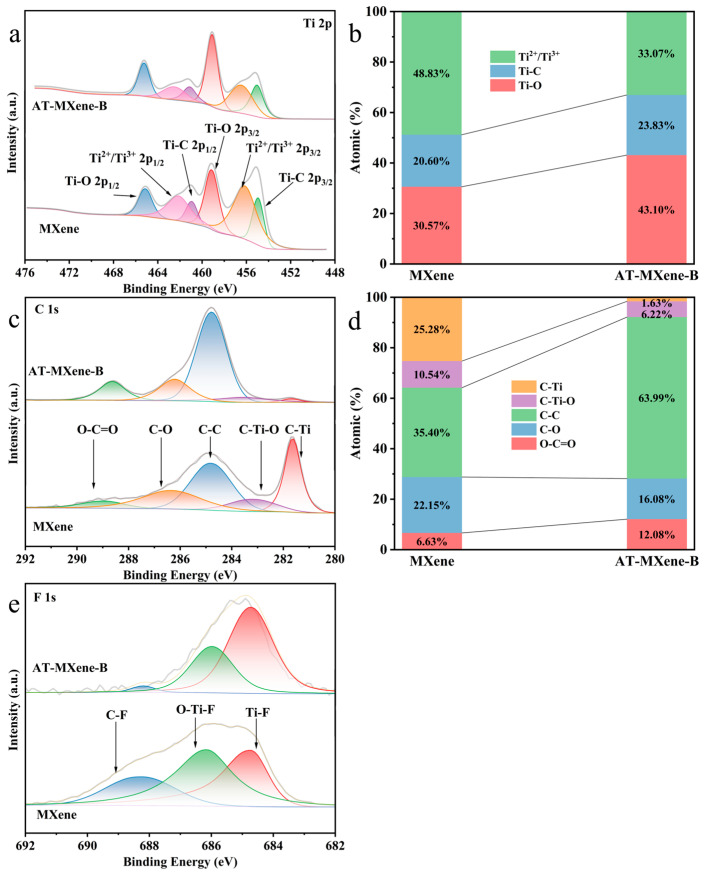
XPS spectrum of MXene and AT-MXene-B: the fine spectrum of (**a**) Ti 2p, (**c**) C 1s, and (**e**) F 1s; XPS peak fitting results for the fine spectrum of (**b**) Ti 2p, and (**d**) C 1s.

XPS spectra reveal the surface chemical changes in MXene induced by alkali treatment. During the KOH-treatment process, the Ti–C bonds of MXene undergo dissociation and cleavage, causing their proportion to plummet from 25.28% to 1.63% (Figure 2c,d). The released free carbon atoms undergo in situ rearrangement on the surface, increasing the proportion of C–C bonds from 35.40% to 63.99%. Meanwhile, the titanium atoms detached from the carbon framework are oxidized and converted into more stable TiO_2_ or oxygen-rich titanium species, which corresponds to a marked rise in the proportion of Ti–O bonds in the Ti 2p spectra (Figure 2a,b) from 30.57% to 43.10%. In addition, as shown in Figure 2e, the almost disappearance of the C–F bonds further confirms the extensive removal of surface F-terminal groups by the alkali treatment, which coincides with the EDS analysis results (Table S1).

**Figure 3:**
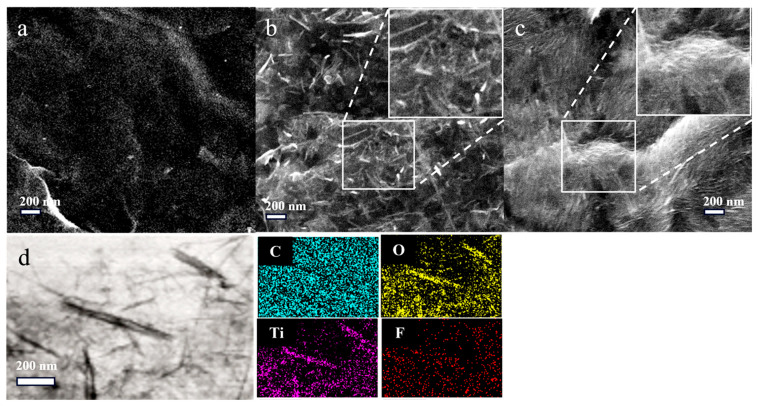
SEM images of (**a**) MXene, (**b**) AT-MXene-A, and (**c**) AT-MXene-B. (**d**) TEM images of AT-MXene-B, along with the corresponding EDS maps for C, O, Ti, and F.

The change in the microstructure of Ti_3_C_2_T_x_ MXene after KOH treatment was observed with SEM imaging. As shown in Figure 3a, pristine MXene displays a characteristic dense layered stacking structure with a relatively smooth surface, suggesting tight interlayer bonding that hinders the penetration and diffusion of electrolyte ions. Following the KOH treatment, both AT-MXene-A (Figure 3b) and AT-MXene-B (Figure 3c) exhibited retention of their flake morphology, whilst displaying some fibrous nanostructures on their surfaces. These phenomena can be attributed to the in situ generation of TiO_2_ nanorods or nanowires due to partial oxidation of Ti in the MXene in the alkaline environment. In comparison with the nanorods observed in AT-MXene-A, those in AT-MXene-B are longer and more densely packed. This suggests that as the KOH treatment time increases, more Ti elements in MXene undergo oxidation, leading to the formation of longer TiO_2_ nanowires. As shown in Figure 3d, a number of TiO_2_ nanowires were formed on the surface of the alkali-treated MXene, exhibiting an intertwined distribution. The EDS mapping reveals a pronounced co-enrichment of Ti and O elements in the nanowire region. Notably, the original 2D layered outline of MXene can still be observed in the TEM image, suggesting that the oxidation process is a localized partial oxidation rather than a complete structural collapse. Consequently, a hierarchical structure composed of a MXene framework and TiO_2_ nanowires is formed. Such a structure can effectively suppress the re-stacking of nanosheets and create more ion transport channels, which benefits its electrochemical capacitive performance.

Moreover, as demonstrated in Table S1, there was a significant decrease in the F content from 5.97 at% to 0.91 at%, accompanied by an increase in the O content from 21.34 at% to 46.55 at% and the decrease in Ti content from 56.43 at% to 25.83 at%, as the KOH treatment progressed for 24 h. This indicates that the -F groups were effectively removed, concomitant with the generation of the 1D TiO_2_ nanostructures. Concurrently, the C content increased from 11.79 at% to 22.87 at%, reflecting the layer exfoliation and structural reconstruction processes. Moreover, the introduction of the K element in AT-MXene-B (Figure 1e) suggests the K^+^ intercalation or retention within the layered MXene structure after the KOH treatment and its content is 2.59 at% (Table S1). The 1D TiO_2_/MXene composite products are totally different from the recent work on the similar alkaline treatment of Ti_3_C_2_T_x_ with KOH solution, where such 1D TiO_2_ nanostructures were absent in their products [7]. This could be due to the low concentration of KOH solution and low temperature used in their work. In summary, the KOH treatment resulted in the transformation of Ti_3_C_2_T_x_ MXene from a dense layered structure to a surface-distributed fibrous structure. This transformation was accompanied by a synergistic regulation of surface groups and hierarchical composite structure. Such changes in Ti_3_C_2_T_x_ MXene were believed to facilitate ion diffusion and electrochemical reactions.

### 3.2. Regulation of MXene Electrochemical Performance via the Synergistic Effect of KOH Treatment of MXene and Iron-Ion-Containing Electrolytes

**Figure 4:**
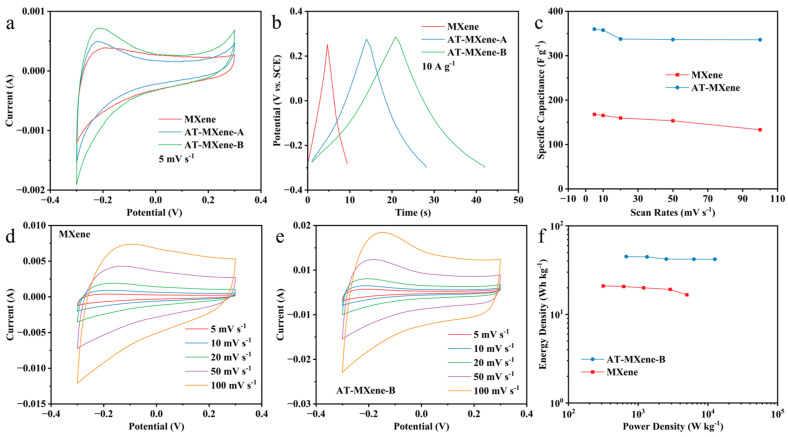
Electrochemical behavior of pristine MXene and KOH-treated MXene in 1M H_2_SO_4_: (**a**) CV curve at a scan rate of 5 mV s^−1^; (**b**) GCD curve at a current density of 10 A g^−1^; CV curves at different scan rates of (**d**) MXene and (**e**) AT-MXene-B; (**c**) the dependence of specific capacitance on the scan rate and (**f**) Ragone plots of MXene and AT-MXene-B.

As shown in Figure 4a, the CV curve of MXene bears a close resemblance to that of MXene in the literature [27], with the capacitance being primarily attributed to the alteration in titanium oxidation state. In contrast, the CV curve area of either AT-MXene-A or AT-MXene-B was significantly enlarged. A substantial enhancement in current response was observed in the range of approximately −0.3 V to −0.2 V, particularly for AT-MXene-B. This behavior is attributed to the fact that KOH treatment effectively regulates MXene surface groups, reducing the content of -F groups and promoting the generation of oxygen-containing active groups. Concurrently, 1D TiO_2_ nanostructures are formed in situ on the surface, thereby introducing more electrochemically active sites on MXene surface, preventing its re-stacking and promoting the electrochemical reactions for capacitance. As suggested previously, oxygen-groups on MXene contribute more capacitance than F-terminal group, and they operate as active sites for the adsorption of hydrogen ions for energy storage reaction [28]. As shown in Figure 4b, the GCD curves illustrate substantial variances in the capacitive performance of samples. Pristine Ti_3_C_2_T_x_ MXene exhibits a specific capacitance of 78.17 F g^−1^ and its GCD curve demonstrates an approximate linear triangular profile. After 12 h alkaline treatment, the specific capacitance of the resulting AT-MXene-A was significantly enhanced to 234.33 F g^−1^. Further increasing KOH-treatment time to 24 h led to a further augment to 350.33 F g^−1^ (AT-MXene-B). Moreover, it exhibits nonlinear charging and discharging characteristics with a region of gentle slope appearing in the curves, which reflects the involvement of Faradaic reactions during the GCD process. As shown in Figure 4d,e, the CV current increased with the scan rate for both Ti_3_C_2_T_x_ MXene and AT-MXene-B samples, and the dependence of the corresponding specific capacitance on the scan rate was plotted as Figure 4c. Clearly, all the capacitances of AT-MXene-B are much larger than those for pristine Ti_3_C_2_T_x_ MXene, indicating that alkaline treatment of MXene significantly enhances its specific capacitance.

In 1M H_2_SO_4_, the AT-MXene-B electrode exhibits markedly enhanced energy storage capability over the entire power density range compared with pristine MXene, as shown in Figure 4f. Specifically, AT-MXene-B achieves an energy density of 44.97 Wh kg^−1^ at a power density of 674.58 W kg^−1^, whereas pristine Ti_3_C_2_T_x_ MXene delivers only 20.68 Wh kg^−1^ under similar conditions. Even when the power density increases to approximately 12,600 W kg^−1^, AT-MXene-B still maintains an energy density above 42 Wh kg^−1^, corresponding to an energy retention of over 90%, which is significantly higher than that of pristine Ti_3_C_2_T_x_ MXene. These results indicate that the alkali treatment of MXene not only enhances the energy density but also maintains excellent energy retention capability at high power output. Its improved Ragone performance can be ascribed to the regulated surface chemical groups and hierarchical composite structure resulting from the alkaline treatment of Ti_3_C_2_T_x_ MXene. These features promote ion transport and expose more electrochemically active sites for charge storage.

**Figure 5:**
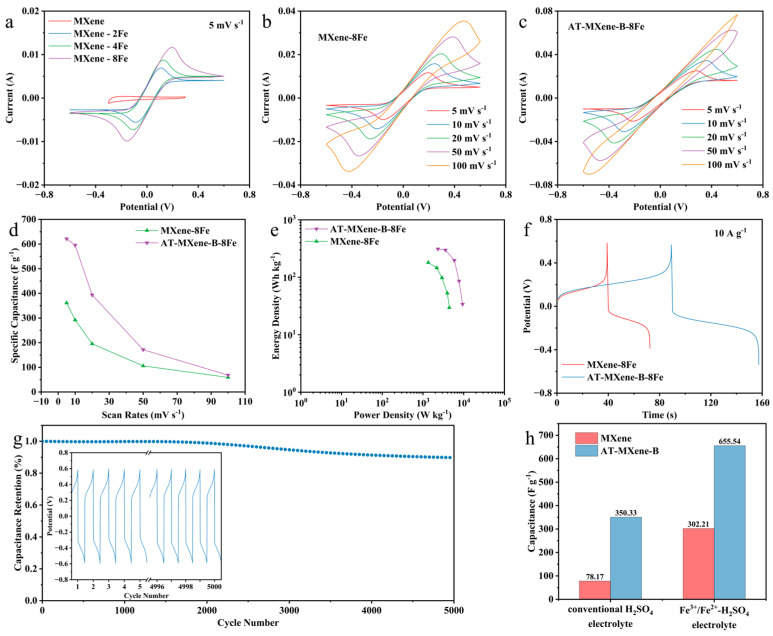
(**a**) CV curves of MXene in 1 M H_2_SO_4_ containing different concentrations of Fe^3+^/Fe^2+^ at a scan rate of 5 mV s^−1^; CV curves at different scan rates of (**b**) MXene-8Fe and (**c**) AT-MXene-B-8Fe; (**d**) the dependence of specific capacitance of MXene-8Fe and AT-MXene-B-8Fe at different scan rates and (**e**) their Ragone plots; (**f**) GCD curves of MXene-8Fe and AT-MXene-B-8Fe at a current density of 10 A g^−1^. (**g**) Cycling stability of AT-MXene-B-8Fe at a current density of 10 A g^−1^. (**h**) The capacitance of MXene and AT-MXene-B in 1 M H_2_SO_4_ and 0.8 M Fe^2+^/Fe^3+^-H_2_SO_4_ electrolyte.

As illustrated in Figure 5a, all the MXene-based electrodes exhibit a pair of distinct redox peaks in the iron-ion electrolyte, which are attributed to the electron transfer process of the Fe^2+^/Fe^3+^ redox couple at the electrode interface, as described in Equation (5). Specifically, the anodic peak during the forward scan represents the oxidation of Fe^2+^ to Fe^3+^, while the cathodic peak in the reverse scan corresponds to the reduction of Fe^3+^ back to Fe^2+^.

(5) $${Fe}^{3+}+e^{-}⇌{Fe}^{2+}$$

In an acidic aqueous electrolyte containing Fe^3+^/Fe^2+^, the reaction sequence of the active species is primarily governed by their oxidizing and reducing abilities. Because the Fe^3+^/Fe^2+^ couple possesses a relatively high and reversible redox activity, pseudocapacitive reactions preferentially occur during the cyclic voltammetry (CV) scan. During the forward scan (potential gradually increasing), the electrode undergoes an oxidation reaction: Fe^2+^ in the system first loses electrons and is oxidized to Fe^3+^, corresponding to the anodic oxidation peak in the CV curve. As the potential rises further, if it exceeds the water stability window in the acidic electrolyte, the oxygen evolution reaction (ORR) may also occur. Therefore, the positive potential of the operation window is set to be 0.6 V, which is far less than that of the ORR (1.23 V vs. RHE). Conversely, during the reverse scan, Fe^3+^, owing to its strong oxidizing nature, preferentially accepts electrons and is reduced to Fe^2+^, producing a distinct cathodic reduction peak. When the potential shifts further negative, H^+^ may participate in reduction reactions, and the hydrogen evolution reaction (HER) may take place. Due to the presence of high concentration of iron ions in the electrolyte, HER hardly occurred within the negative potential limit of the operation voltage window (−0.6 V) in this work. Therefore, throughout the entire CV scan within the voltage window of −0.6~0.6 V, the reversible Fe^3+^/Fe^2+^ redox couple dominates the major Faradaic pseudocapacitive behavior without the occurrence of both ORR and HER. This could also be verified by its excellent long-term cycling stability of the electrochemical system of AT-MXene-B in 0.8 M Fe^2+^/Fe^3+^-H_2_SO_4_ electrolyte (AT-MXene-B-8Fe) operated within the potential window, as shown in Figure 5g. In fact, the multi-cyclic stability is a crucial parameter for evaluating the practical viability of supercapacitors. As can be seen from Figure 5g, the AT-MXene-B-8Fe system retains its capacitance as high as approximately 90% of its initial value after 5000 GCD cycles.

In common H_2_SO_4_ electrolyte, it is noteworthy that the stable voltage range of MXene is comparatively constrained compared to that in 1 M H_2_SO_4_ electrolyte containing Fe^2+^/Fe^3+^, as shown in Figure 5a. Actually, widening its potential window results in an irreversible electrochemical reaction of the MXene electrode material (Figure S1). Therefore, the stable potential range of pristine Ti_3_C_2_T_x_ MXene in 1 M H_2_SO_4_ solution in Figure 4 is set to be from −0.3 V to 0.3 V, which contrasts sharply with its wide voltage range of −0.6 V to 0.6 V in the redox-active electrolyte containing Fe^2+^/Fe^3+^. Besides the widened potential window in the redox-active electrolyte, the CV curve area of MXene in 0.2 M Fe^2+^/Fe^3+^-H_2_SO_4_ electrolyte (MXene-2Fe) is significantly enlarged, and distinct redox peaks appear in the range from −0.1 to 0.2 V, indicating the introduction of reversible Faradaic reactions centered on Fe^2+^/Fe^3+^ in the system. This change is primarily attributed to the regulation of the surface chemical state of Ti_3_C_2_T_x_ MXene by KOH-treatment: most F- groups were removed, oxygen-containing active groups increased, and a 1D TiO_2_ nanostructure was formed in situ on the surface, thereby providing more active sites with affinity for iron ions, enhancing their interfacial adsorption and charge transfer capabilities. Moreover, an increase in the concentration of Fe^2+^/Fe^3+^ in the electrolyte results in a gradual strengthening of the redox peak current. According to the previous work [19], a high concentration of Fe^3+^/Fe^2+^ redox additive of up to 0.8 M could exist stably in normal sulfate acid electrolyte. Among these electrolytes, MXene-8Fe demonstrated the most pronounced redox response, suggesting its superior electrochemical capacitive performance. Therefore, 0.8 M Fe^2+^/Fe^3+^-H_2_SO_4_ electrolyte is determined to be the optimized electrolyte for pairing with the MXene electrode in this work. The findings were further validated by GCD testing. As shown in Figure 5f, at 10 A g^−1^, AT-MXene-B-8Fe exhibited a great increase in discharge time and its specific capacitance is calculated to be 655.54 F g^−1^, which is double that of MXene-8Fe (302.21 Fg^−1^). And this value is more than eight times that of Ti_3_C_2_T_x_ MXene in common H_2_SO_4_ electrolyte (Figure 4b), confirming the effectiveness of the proposed strategy for synergistic regulation of the MXene electrode material and redox activity of electrolyte. It is noted that this specific capacitance is remarkably larger than most capacitance values for the various MXene materials in the literature, as can be seen in Table 1. Additionally, the flatter and longer voltage plateau in its GCD curves suggests an even more significant contribution from Faradaic reactions. In fact, as shown in Figure 5h, the respective contributions of the MXene surface modification (the formation of TiO_2_ on Ti_3_C_2_T_x_) and the addition of Fe^2+^/Fe^3+^ additive to electrolyte to the capacitance enhancement of the electrochemical system could be clarified by the control experimental results. Similar to those in conventional H_2_SO_4_ electrolyte, the CV currents increase with the scan rate for both Ti_3_C_2_T_x_ MXene and AT-MXene-B, as shown in Figure 5b and Figure 4c. It can be seen that, at the same scan rate, the CV current of AT-MXene-B is much larger than that of pristine MXene. The dependence of the corresponding specific capacitance on the scan rate was plotted in Figure 5d. Evidently, all the capacitances of AT-MXene-B and MXene decreased with the increasing scan rate and the capacitance of AT-MXene-B was always larger than that of MXene at the same scan rate. The capacitance difference between them changed with the scan rate, and the largest one was achieved at the smallest scan rate (5 mV s^−1^), implying the diffusion-controlled electrochemical reaction in the system with redox electrolyte.

As illustrated in Figure 5e, the AT-MXene-B-8Fe system achieves a remarkably high energy density of nearly 310 Wh kg^−1^ at a power density of 2330 W kg^−1^, substantially surpassing that of the MXene-8Fe system (145 Wh kg^−1^). This dramatic increase demonstrates the effectiveness of Fe^3+^/Fe^2+^ redox couples in contributing additional pseudocapacitance through reversible Faradaic reactions, thereby greatly boosting the overall energy density of the electrochemical system. More importantly, the AT-MXene-B-8Fe consistently delivers higher energy densities than MXene-8Fe across the entire power density range, indicating that the alkali-treated MXene enables more efficient utilization of redox-active ions.

### 3.3. The Synergistic Energy Storage Mechanism of KOH-Treated MXene in Iron-Ion-Containing Electrolytes

**Figure 6:**
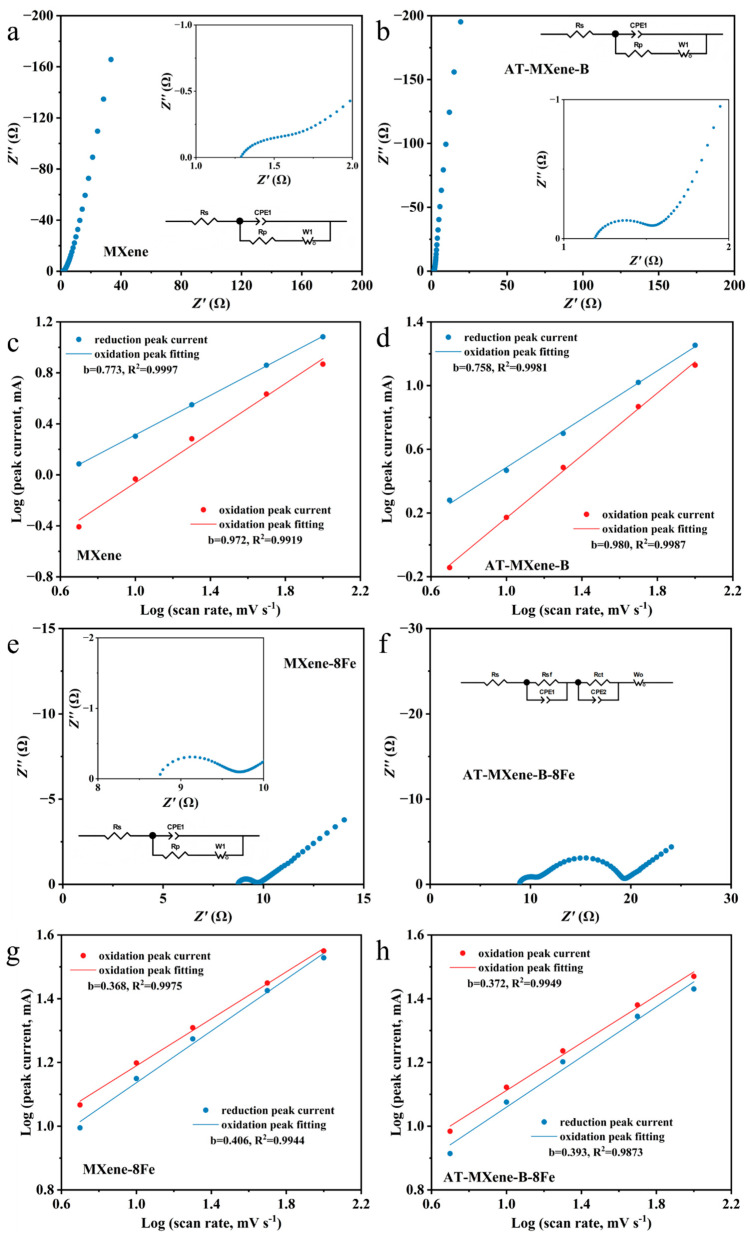
The Nyquist plot for (**a**) MXene, (**b**) AT-MXene-B, (**e**) MXene-8Fe, and (**f**) AT-MXene-B-8Fe, accompanied by an enlarged view of the high-frequency region and an equivalent circuit diagram; logarithmic relationship between peak current and scan rate for (**c**) MXene, (**d**) AT-MXene-B, (**g**) MXene-8Fe, and (**h**) AT-MXene-B-8Fe.

The EIS test provides further elucidation on the mechanism behind the performance enhancement from a kinetic perspective. The impedance spectra were fitted using equivalent circuits (Figure 6a,b,e), where R_s_ represents the equivalent series resistance, R_ct_ corresponds to the charge transfer resistance at the electrode/electrolyte interface, CPE denotes the constant phase element accounting for non-ideal capacitive behavior, and W_o_ describes the Warburg diffusion associated with ion transport [36,37,38].

In common H_2_SO_4_ electrolyte, the Nyquist plot of Ti_3_C_2_T_x_ MXene (Figure 6a) exhibits a smaller semicircle in the high-frequency region than that of AT-MXene-B (Figure 6b), indicating a lower charge transfer resistance. Meanwhile, in the low-frequency region, the AT-MXene-B curve is closer to vertical, indicating that its ion diffusion process is closer to ideal capacitive behavior. This phenomenon is attributed to the restructuring of the surface physicochemical structure by KOH treatment, and the formation of TiO_2_ nanowires on the Ti_3_C_2_T_x_ MXene surface, which provide wider diffusion channels and more electrochemically active sites for active ions. In the redox-active electrolyte, the larger semicircular diameter of MXene-8Fe (inset in Figure 6e) than that of its counterpart Ti_3_C_2_T_x_ MXene system (inset in Figure 6a) indicates a substantial increase in charge transfer resistance. Concurrently, the curve slope in the low-frequency region is reduced. This phenomenon can be attributed primarily to the redox reactions of the additional Fe^2+^/Fe^3+^ ions at the electrode/electrolyte interface, which endow the system with high capacitive performance along with a strong dependence on the ion diffusion kinetics. As shown in Figure 6e, the Nyquist plot of MXene-8Fe consists of a typical “single compressed semicircle + low-frequency Warburg line,” indicating that the system is primarily dominated by a single interfacial electrochemical reaction process and possesses a single primary time constant. This suggests that the energy storage mechanism of this system is majorly governed by pseudocapacitive behavior, which is controlled by ion diffusion.

In contrast, the Nyquist plot of AT-MXene-B-8Fe exhibits a distinct “broadened semicircle” or even a “double-arc trend” in the high-to-mid-frequency range (Figure 6f), indicating a more complex interfacial reaction process. The equivalent circuit of this system necessitates the incorporation of additional interfacial elements for effective description. Among these, similar to previous reports [39], additional Rsf and CPE1 corresponding to the resistance and capacitive responses of the active layers (such as the oxygen-enriched layer and iron ion coordination layer) formed on the Ti_3_C_2_T_x_ MXene surface after alkali treatment are added, while Rct and CPE2 related to the charge transfer process between the electrode body and the electrolyte remain in the equivalent circuit. The emergence of this dual-parallel structure indicates the presence of two distinct time constants in the electrochemical system, corresponding to the “surface-modified layer response” and the “interfacial charge transfer process”. This suggests that the electrochemical reaction of the entire AT-MXene-B-8Fe system has evolved to a multi-interface synergistic process from a single-interface process for the MXene-8Fe system.

In order to quantitatively analyze the energy storage kinetics, the CV curves of the electrochemical systems obtained at different scan rates were fitted to power-law relationships. The relationship between peak current and scan rate follows the power-law $i=av^{b}$ [18,40], where the b-values provide insight into the dominant kinetic process. As shown in Figure 5c, the b-values for the oxidation and reduction peaks of Ti_3_C_2_T_x_ MXene are 0.972 and 0.770, respectively; in the meanwhile, the values for the oxidation peak and reduction peak of AT-MXene-B are 0.980 and 0.758 (Figure 6d). The minor discrepancies between them suggest that their energy storage processes are governed by surface capacitance of the electrode materials. In the iron-ion-containing electrolyte, the b-values of the oxidation and reduction peaks for MXene-8Fe are 0.368 and 0.406, respectively, as shown in Figure 6g, while those for AT-MXene-B-8Fe are 0.372 and 0.393 (Figure 6f). Such minor discrepancies between them mean that both electrochemical systems are synergistically controlled by interfacial reactions, despite the change in the Ti_3_C_2_T_x_ MXene electrode materials in the surface chemical properties by KOH treatment and the formation of a hierarchical structure of TiO_2_/MXene composite. These demonstrate unequivocally that the introduction of mixed iron ions in the common electrolyte significantly enhances the insertion/extraction process of ions within the electrode, thereby shifting the energy storage mechanism from being dominated by surface adsorption to the diffusion of electrolyte ions.

**Figure 7:**
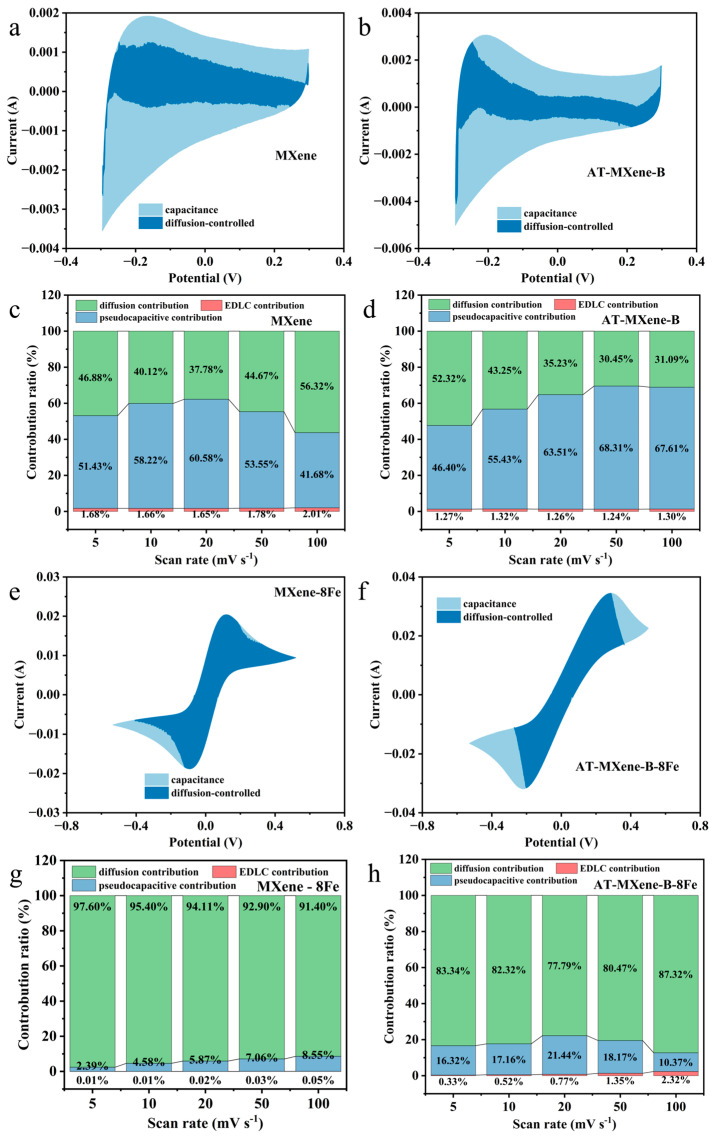
Diffusion-controlled capacitance of (**a**) MXene, (**b**) AT-MXene-B, (**e**) MXene-8Fe, and (**f**) AT-MXene-B-8Fe at 20 mV s^−1^, and the proportion of pseudocapacitive contribution of (**c**) MXene, (**d**) AT-MXene-B, (**g**) MXene-8Fe, and (**h**) AT-MXene-B-8Fe at different scan rates.

To further quantify the charge storage contributions, the current response was deconvoluted into capacitive ($k_{1}v$) and diffusion-controlled ($k_{2}v^{1/2}+k_{3}v^{3/2}$) components based on $i\left(V\right)=k_{1}v+k_{2}v^{1/2}+k_{3}v^{3/2}$ [41,42]. A thorough examination of the contributions to the total capacitance can provide a more precise insight into the relative proportions of different energy storage mechanisms. In common H_2_SO_4_ electrolyte, for AT-MXene-B, the diffusion-controlled contribution was estimated to be 52.32% at 5 mV s^−1^, as shown in Figure 7d. However, as the scan rate increased to 100 mV s^−1^, this proportion decreased to 31.09%. Consequently, the pseudocapacitive contribution exhibited an increase from 46.40% to 67.61%, while the EDLC contribution remained at a consistently negligible level. In contrast, for pristine Ti_3_C_2_T_x_ MXene, the diffusion contribution fluctuates between 37.78% and 56.32%, and the pseudocapacitance contribution varies accordingly between 41.68% and 60.58%, despite the slightly higher EDLC contribution, as shown in Figure 7c. It can be seen that, only at the largest scan rate in the work (100 mV s^−1^), the dominant contribution switches from pseudocapacitance to the diffusion-controlled part. These results indicate that AT-MXene-B exhibits strong ion diffusion-controlled characteristics at low scan rates, while gently shifting toward surface pseudocapacitance dominance at high scan rates, demonstrating excellent rate capability and kinetic adaptability.

In contrast, in the iron-ion-containing electrolyte, for the MXene-8Fe system (Figure 7g), the diffusion-controlled contribution consistently dominated across all the scan rates (91.40–97.60%), while the pseudocapacitive contribution was only 2.39–8.55%. This discrepancy further substantiates the fact that iron ions efficaciously induce pseudocapacitive reactions and promote the profound diffusion of electrolyte ions into the MXene electrode, thereby considerably enhancing the utilization of active sites. Meanwhile, for AT-MXene-B-8Fe (Figure 7h), the diffusion contribution diminished a little to 77.79–87.32%, while the pseudocapacitive contribution augmented substantially to 10.37–21.44%. These results indicate that alkali treatment effectively modulates the surface properties of the MXene electrode material, altering the energy storage mechanism of the corresponding electrode/electrolyte system from being almost solely diffusion-controlled to being jointly dominated by diffusion and pseudocapacitance. Such a change in mechanism benefits its energy storage application.

## 4. Conclusions

The present work investigated the synergistic effects of KOH treatment of Ti_3_C_2_T_x_ MXene electrode materials and the utilization of redox-active electrolytes on the electrochemical behavior of the resulting energy storage system. Results indicate that KOH treatment removes most F- terminal groups and introduces -O/-OH terminal groups on MXene and in situ grows 1D TiO_2_ nanostructures, thereby modifying its surface chemical properties and geometric structure. The addition of Fe^2+^/Fe^3+^ to its conventional sulfuric acid electrolyte results in the manifestation of distinct pseudocapacitive behavior within the electrode in the electrochemical system. The synchronous and synergistic regulation of the electrode and electrolyte (AT-MXene-B-8Fe) leads to a significant increase in the specific capacitance to 655.54 F g^−1^, and a shift in the energy storage mechanism from being solely diffusion-controlled to being jointly controlled by diffusion and surface reactions. Accordingly, the equivalent circuit of the electrochemical system has evolved from a single-interface model to a dual-interface model. The present study has demonstrated that this novel synergistic strategy proposed in this work can significantly enhance the electrochemical capacitive performance of the MXene-based energy storage system and potentially be applied in other electrochemical systems.

## Figures and Tables

**Table 1:** Capacitive performances of the Ti_3_C_2_T_x_ MXene electrode materials.

Materials	Conditions	Electrolyte	Gravimetric Capacitance
This work	10 A g^−1^	1 M H_2_SO_4_ + 0.8 M Fe^2+^/Fe^3+^	655.54 F g^−1^
KOH etched Ti_3_C_2_T_x_ [7]	2 A g^−1^	1 M H_2_SO_4_	368.1 F g^−1^
NaOH–Ti_3_C_2_T_x_ [29]	1 A g^−1^	1 M H_2_SO_4_	254 F g^−1^
Ti_3_C_2_T_x_/I_2_ [30]	1 mV s^−1^	1 M H_2_SO_4_	293 F g^−1^
I-Ti_3_C_2_ MXene [31]	2 A g^−1^	1 M H_2_SO_4_	124 F g^−1^
Ti_3_C_2_T_x_ pillared by hydrazine [32]	2 mV s^−1^	1 M H_2_SO_4_	250 F g^−1^
holey-Ti_3_C_2_T_x_ framework [33]	10 mV s^−1^	3 M H_2_SO_4_	359.2 F g^−1^
Hierarchical porous MXene [34]	2 A g^−1^	3 M H_2_SO_4_	539 F g^−1^
Porous Ti_3_C_2_T_x_ [35]	1 A g^−1^	3 M H_2_SO_4_	498.3 F g^−1^
Ti_3_C_2_ MXene nanomesh [35]	1 A g^−1^	6 M KOH	235 F g^−1^

## Data Availability

The original contributions presented in the study are included in the article; further inquiries can be directed to the corresponding author.

## Supplementary Materials

The following supporting information can be downloaded at: https://www.mdpi.com/article/10.3390/nano16110671/s1, Figure S1: CV curve of MXene at a scan rate of 5 mV s^−1^ from −0.6 V to 0.6 V; Table S1: Elemental contents from EDS of various MXene samples.
