# Supplementary material for: Synergistically Enhancing Capacitive Performance of Ti3C2Tx MXene via Building Hierarchical Structure of TiO2 Nanowire/MXene Composites and Utilizing Iron-Ion-Based Redox-Active Electrolytes
Source: Nanomaterials (Basel). 2026 May 27;16(11):671. doi: 10.3390/nano16110671 (PMC13257605; doi:10.3390/nano16110671)
Supplement: Supplementary file 1 [file nanomaterials-16-00671-s001.zip › nanomaterials-4312965-supplementary.pdf]

nanomaterials

Article

Synergistically Enhancing Capacitive Performance of $Ti_3C_2T_x$ MXene via Building Hierarchical Structure of TiO_2 Nanowire/MXene Composites and Utilizing Iron-Ion Based Redox Active Electrolytes

Xiaohan Wang [1] and Xusheng Du [1,*]

[1] Institute of Advanced Wear & Corrosion Resistant and Functional Materials, School of Chemistry and Materials, Jinan University, Guangzhou 510632, China
* Correspondence: xdusydjn@email.jnu.edu.cn

Support information

Table S1. Elemental contents from EDS of various MXene samples

Sample	Ti Content	O Content	C Content	K Content	F Content	Cl Content
MXene	56.43 at %	21.34 at %	11.79 at %	\	5.97 at %	4.46 at %
AT-MXene-A	31.82 at %	42.15 at %	18.50 at %	3.77 at %	1.97 at %	1.79 at %
AT-MXene-B	25.83 at %	46.55 at %	22.87 at %	2.59 at %	0.91 at %	1.26 at %

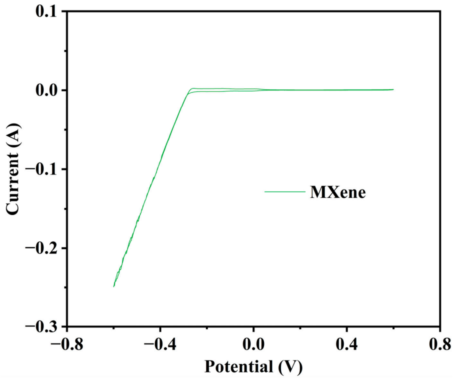

Figure S1. CV curve of MXene at a scan rate of 5 mV s^{-1} from -0.6 V to 0.6 V
